# Distinct Neural Resource Involvements but Similar Hemispheric Lateralization Patterns in Pre-Attentive Processing of Speaker’s Identity and Linguistic Information

**DOI:** 10.3390/brainsci13020192

**Published:** 2023-01-23

**Authors:** Shuqi Yin, Lang Xie, Yunxiao Ma, Keke Yu, Ruiming Wang

**Affiliations:** Philosophy and Social Science Laboratory of Reading and Development in Children and Adolescents, Ministry of Education, & Center for Studies of Psychological Application, School of Psychology, South China Normal University, Guangzhou 510631, China

**Keywords:** speech perception, identity information, linguistic information, mismatch negativity (MMN)

## Abstract

The speaker’s identity (who the speaker is) and linguistic information (what the speaker is saying) are essential to daily communication. However, it is unclear whether and how listeners process the two types of information differently in speech perception. The present study adopted a passive oddball paradigm to compare the identity and linguistic information processing concerning neural resource involvements and hemispheric lateralization patterns. We used two female native Mandarin speakers’ real and pseudo-Mandarin words to differentiate the identity from linguistic (phonological and lexical) information. The results showed that, in real words, the phonological-lexical variation elicited larger MMN amplitudes than the identity variation. In contrast, there were no significant MMN amplitude differences between the identity and phonological variation in pseudo words. Regardless of real or pseudo words, the identity and linguistic variation did not elicit MMN amplitudes differences between the left and right hemispheres. Taken together, findings from the present study indicated that the identity information recruited similar neural resources to the phonological information but different neural resources from the lexical information. However, the identity and linguistic information processing did not show a particular hemispheric lateralization pattern at an early pre-attentive speech perception stage. The findings revealed similarities and differences between linguistic and non-linguistic information processing, contributing to a better understanding of speech perception and spoken word recognition.

## 1. Introduction

Listeners usually need to process two types of information, linguistic and non-linguistic, during speech perception [1,2]. Linguistic information consists of words’ phonological, lexical, and semantic information, conveying what the speaker is saying. The non-linguistic information refers to speakers’ information, such as the speakers’ identities (who the speaker is) and accents. How linguistic information is processed is a traditional issue in speech perception and spoken word recognition studies [3,4,5,6,7]. Recently, researchers have highlighted the role of non-linguistic information in spoken word recognition [8,9,10,11]. However, an important and unresolved question is whether and how non-linguistic information, especially identity information processing, differs from linguistic information processing. The present study discussed the issue by conducting an event-related potential (ERP) experiment with the mismatch negativity (MMN) component. 

### 1.1. Linguistic and Non-Linguistic Information

Traditional spoken word recognition models and speech perception studies have discussed how listeners represent and process the linguistic information from the speech signals (e.g., the TRACE Model [12]; the Neighborhood Activation Model [13]; [14,15]). For example, the TRACE Model hypothesizes three levels of representation for spoken words, including the features level, phonemes level, and words level. The features level consists of the acoustic features of vowels and consonants (e.g., burst, friction) (and perhaps the pitch features of lexical tones in tonal languages only [16]). The phonemes level stores the specific vowels and consonants (e.g., /a/, /t/) (and perhaps lexical tones in tonal languages only). The words level includes the input word and its related words (e.g., cat, hat). The model assumes interactions between the different levels of processing in spoken word recognition. The lower levels could activate the higher levels. The higher levels could also give feedback and influence the lower levels’ processing.

However, recent studies pointed out that non-linguistic information could also be encoded and represented [17,18,19]. It could play a role in spoken word recognition [20,21]. The Episodic view proposes that the speech signals are not immediately mapped onto the abstract lexical representations but leave a unique memory trace [22,23]. The memory trace includes not only linguistic but also non-linguistic information. Listeners could encode non-linguistic information as a part of lexical representation [9]. Therefore, non-linguistic information may affect spoken word recognition. 

For example, the “Single-voice dominance effect” refers to the phenomenon that speaker variations hinder speech processing [22,24]. The multiple speakers’ speech stimuli may reduce or impede the priming effect of phonological and lexical information on spoken word recognition to a certain extent [24,25]. The “Speaker Familiarity Effect” also provided evidence for the view. However, it suggested a positive role of identity information [26,27]. Being familiar with a speaker’s voice, such as a friend’s or spouse’s, can facilitate the listener’s word repetition performance [27]. The two effects suggested unfamiliar speakers’ identity information may interfere with speech perception and comprehension. However, once listeners become familiar with the specific identity information and store it in the long-term memory, they could perform better.

### 1.2. The Identity vs. Linguistic Information Processing

Although many studies indicated the identity information’s role in spoken word recognition, it is unclear whether and how identity information processing differs from linguistic information processing. Zhang et al. (2016) examined the interaction between lexical tone and speakers’ identity processing [28]. They found that the processing of lexical tones and speakers’ identities interacted at 500–800 ms after auditory words onsets. Moreover, the interaction was asymmetrical. The unattended speaker variations affected the linguistic processing to a larger degree than the unattended linguistic variations affected the identity processing. The results indicated that the identity and linguistic information processing would be distinct. However, the study did not directly compare the two types of information processing. 

Some studies explored the issue more directly by comparing the identity with more detailed linguistic information, phonological, or lexical information. The phonological information refers to different phonemes, such as vowels and consonants. The lexical information refers to the words’ auditory forms and meanings, which is important to lexical access in spoken word recognition [12,29]. Tuninetti et al. (2017) found that the identity and phonological information evoked similar MMN amplitude, indicating that the two types of information were processed similarly [30]. Di Dona et al. (2022) also found a similar MMN amplitude between identity and phonological information processing [31]. However, the two types of information processing differed at the later processing stage, as the identity variations elicited larger late discriminative negativity (LDN) amplitudes than the phonological variations. Taken together, the identity information processing may be similar to the phonological information processing at the early MMN time window while different at the later LDN time window. 

Does the identity information processing differ from the lexical information? An early study by Knösche et al. (2002) suggested that identity and linguistic information processing would be similar [32]. They did not find N100 m (the magnetic equivalent of the MMN) amplitude differences between the identity and linguistic information variations. However, they adopted real German words as the materials and did not differentiate the phonological from the lexical information, as phonological information variations occur with lexical information variations in real words simultaneously. Therefore, the issue remains unclear. 

The acoustic hypothesis considers that the hemispheric lateralization of auditory processing depends on sounds’ acoustic variations. The temporal variation processing mainly occurs in the left hemisphere, while the spectral variation processing occurs mainly in the right hemisphere [33,34]. As speakers’ identities mainly differ in the F0 features (spectral variation), their processing would lateralize to the right hemisphere. However, the linguistic information usually varies temporally (e.g., rapidly changing formant transitions characteristic). Its processing would lateralize to the left hemisphere. In contrast, the functional hypothesis considers that sounds’ linguistic functions determine hemispheric lateralization patterns [35,36]. The linguistic information would lateralize to the left hemisphere because of its linguistic function, while the identity information would lateralize to the right hemisphere due to its non-linguistic properties. Regardless of the hypothesis, the processing of linguistic information seems to lateralize to the left hemisphere, while identity information lateralizes to the right hemisphere. 

Previous studies have provided some preliminary evidence for this view. For example, von Kriegstein et al. (2003) found that compared with speech comprehension, the right anterior superior temporal sulcus (STS) and a part of the right precuneus were activated to a larger degree in processing the speaker’s identity processing [37]. However, compared with identity processing, the left middle STS region was activated to a larger degree in speech comprehension. Belin and Zatorre (2003) found that the right anterior STS showed reduced activity when syllables were spoken by the same speaker compared to those spoken by different speakers [38]. Myers and Theodore (2017) exposed participants to two different speakers and asked participants to complete a phonetic categorization task [39]. They found that the right temporoparietal regions were sensitive to speaker variation, whereas the left posterior temporal regions showed sensitivity to phonetic variation. Recently, Overath and Paik (2021) emphasized the importance of the left inferior frontal gyrus for linguistic processing [40]. Schelinski et al. (2022) suggested the significance of the right inferior colliculus for speakers’ identity processing [41]. The above findings indicate that the processing of identity and linguistic information would lateralize to the left and right hemispheres, respectively. 

### 1.3. The Present Study

A speaker’s identity and linguistic information are essential to daily communication. Exploring how the two types of information processing contribute to understanding the mechanism underlying speech perception and spoken word recognition is crucial. However, there remain several significant issues to be solved. Firstly, it needs to be clarified whether identity information processing recruits similar or different neural sources from the linguistic, especially lexical information processing. Although Tuninetti et al. (2017) and Di Dona et al. (2022) found similar identity and phonological information processing, they did not explore the differences between identity and lexical information processing [30,31]. Secondly, previous studies mainly adopted attentive tasks such as the speaker’s identity identification task and the speech perception/comprehension task [28,37,42,43]. The identity identification task requires participants’ attention to the identity information, while the speech perception/comprehension task focuses on the linguistic information. The different attention requirements would affect identity and linguistic information processing detection. Lastly, previous hypotheses and studies suggested that linguistic information processing lateralized to the left hemisphere while identity information processing tended to the right hemisphere. However, most of the evidence came from the functional magnetic resonance imaging (fMRI) studies also adopted attentive tasks as mentioned [28,39,40,41]. It remains unclear whether hemispheric lateralization patterns occur at early pre-attentive speech perception. 

In order to resolve the issues, we adopted the event-related potential (ERP) technique with a passive oddball paradigm to compare the neural resource involvements and hemispheric lateralization patterns in processing the identity and linguistic (phonological and lexical) information. The oddball paradigm is a classical paradigm to detect the pre-attentive processing of speech perception [44,45,46,47]. It contributes to exploring identity and linguistic information processing without attention bias.

For the two specific types of linguistic information, we adopted two real and two pseudo-Mandarin words spoken by two speakers to construct four types of word pairs, i.e., real words with identity variation (speaker A: /su4-liao4/ (means plastic in English)—speaker B: /su4-liao4/ (means plastic in English)), real words with phonological–lexical variation (speaker A: /su4-liao4/ (means plastic in English)—speaker A: /tong2-zhi4/ (means comrades in English)), pseudo-words with identity variation (speaker A: /be2-fai4/—speaker B: /be2-fai4/) and pseudo-words with phonological variation (speaker A: /be2-fai4/—speaker A: /fi1-tei2/). With these word pairs, we could detect distinctions between the identity and phonological information processing by comparing the pseudo-words with identity variation and that with phonological variation. Furthermore, we could examine the differences between identity and lexical information processing by comparing the real words with identity variation and that with phonological–lexical variation.

The oddball paradigm usually elicits the classic ERP component, MMN [44,48]. It is a negative deflection with a frontocentral scalp distribution, peaking approximately 150 and 200 ms after stimuli onset [49]. It is a sensitive indicator to reflect the pre-attentive processing of acoustic and speech variations [49,50]. In ERP studies, the component’s amplitude could reflect the neural resources involved in cognitive processing [51]. Therefore, we focused on the MMNs amplitudes elicited by these different types of stimuli in the present study. 

Based on previous hypotheses and studies, we hypothesized that identity information processing would be similar to phonological information processing but different from lexical information processing concerning neural resource involvements and hemispheric lateralization patterns. Regarding neural resource involvements, we expected to find an interaction between lexicality (real words, pseudo-words) and information (identity information, linguistic information) in MMN amplitudes. In terms of hemispheric lateralization patterns, we expected to find an interaction between lexicality (real words, pseudo-words), information (identity information, linguistic information), and hemisphere (left, right). 

## 2. Methods

### 2.1. Participants

We performed a statistical power analysis using Gpower 3.1.9.7 software [52]. Taking the suggested effect size (*η^2^_p_* = 0.4) in Gpower Manual, a sample size of 21 participants was needed to detect an effect of this size (power = 0.8, *α* = 0.05). Thus, we recruited 24 undergraduate students from South China Normal University to participate in the experiment (9 males, age range: 18–25 years, mean age: 21 years, SD: 1.69). All the participants were native Mandarin speakers. They had normal hearing and (corrected–normal) vision. According to the Edinburgh handedness test [53], they were all right-handed and reported no history of speech, language, neurological disorders, head damage, or mental illness. The participants all signed a consent form before they took part in the experiment and received monetary compensation after the experiment. The Ethics Review Board of South China Normal University approved the study.

### 2.2. Materials

We adopted two real and two pseudo-Mandarin words in the experiment (real words: /tong2-zhi4/ (comrades), /su4-liao4/ (plastic); pseudo-words: /be2-fai4/, /fi1-tei2/). The pseudo-words consisted of Mandarin vowels, consonants, and lexical tones, which conform to Mandarin’s pronunciation rules but do not have meanings in Mandarin. All the words were recorded by two female native Mandarin speakers via Cool Edit Pro 2.1 (http://www.syntrillium.com, accessed on 7 January 2021) at a sampling rate of 44.1 kHz. The words’ duration was between 740 and 860 ms. Their intensity was standardized to 70 dB by the Praat software (http://www.fon.hum.uva.nl/praat/, accessed on 13 January 2021). We used the four words to construct four types of word pairs, i.e., real words with identity variation (RI), real words with phonological-lexical variation (RL), pseudo-words with identity variation (PI), pseudo-words with phonological variation (PP).

To ensure that the materials meet the experimental requirements, we recruited 20 participants, 10 assessing the speakers’ voice similarity and the other 10 assessing the words’ familiarity and intelligibility. All assessments were performed using a 7-point Likert scale from least similar/familiar/intelligible (1 point) to very similar/familiar/intelligible (7 point). These participants did not participate in the later EEG experiment. The results showed that the voice similarity among words spoken by the same speaker (6.28 ± 0.65) was significantly greater than that by different speakers (1.71 ± 0.61) (*t*(9) = 14.922, *p* < 0.001, *d* = 9.948), indicating that the two speakers differed in their voices acoustically. The familiarity and intelligibility of real words (familiarity: 6.63 ± 0.64; intelligibility: 6.55 ± 0.65) were significantly greater than those of pseudo-words (familiarity: 1.40 ± 0.44; intelligibility: 1.30 ± 0.50) (familiarity: *t*(9) = 24.612, *p* < 0.001, *d* = 16.408; intelligibility: *t*(9) = 16.959, *p* < 0.001, *d* = 11.306), indicating that listeners indeed perceived the pseudo-words as pseudo-words. Moreover, there were no significant familiarity and intelligibility differences between the two real words (familiarity: *t*(9) = 0.19, *p* = 0.853, *d* = 0.127; intelligibility: *t*(9) = 0.259, *p* = 0.801, *d* = 0.173) and the two pseudo-words (familiarity: *t*(9) = 1.861, *p* = 0.096, *d* = 1.241; intelligibility: *t*(9) = 0.001, *p* > 0.999, *d* = 0.001). These results ensured that the materials could be used effectively in the study.

### 2.3. Procedure

We adopted a classic passive oddball paradigm in the experiment [54]. There were four blocks in the experiment. Each block contained one type of standard stimuli (96 trials) and one type of deviant stimuli (18 trials). Table 1 shows the standard and deviant stimuli in each block. The standard and deviant stimuli in each block were presented pseudo-randomly, and there were at least three standard stimuli between adjacent deviant stimuli. The stimulus-onset-asynchrony (SOA) between any two stimuli was 1000 ms. There were 15 additional standard stimuli presented to the participants at the beginning of each block to help the participants be familiar with the experiment. The presentation sequence of the four blocks was counterbalanced across the participants. There was a 1-min interval between any two blocks.

The participants were tested individually in a quiet room. The stimuli were presented over headphones at a comfortable sound level. They were instructed to watch a silent movie seriously and ignore the auditory stimuli in the experiment. They did not need to respond to the auditory stimuli. To ensure that the participants focused on the movie, they had to answer five questions about its content after the experiment. The whole experiment lasted about 25 min.

### 2.4. EEG Recording

EEG was recorded using a 64-channel (Ag-AgCl) NeuroScan system (NeuroScan, http://www.neuroscan.com/, accessed on 1 March 2021). The electrodes were positioned following the 10–20 system convention. The reference electrode was placed at the tip of the nose. Supra- and infra-orbitally from the left eye were recorded as the vertical electrooculogram (EOG), and the left versus right orbital rim was recorded as the horizontal EOG. The impedance of each electrode was kept below 5 kΩ. EEG and EOG signals were digitized online at 1000 Hz and band-pass filtered from 0.05 to 100 Hz.

### 2.5. Data Pre-Processing

Off-line signal processing was carried out using Scan 4.5 (NeuroScan, http://www.neuroscan.com/, accessed on 10 June 2021). The reference electrode was first converted to bilateral mastoid (M1 and M2). The interference of the horizontal and vertical eye movements was then eliminated. After that, the data were segmented for a 1000 ms time window, including a 100 ms pre-stimulus baseline. Then, the baseline was corrected. The recorded trials with eye blinks or other activities beyond the range of ±80 μV were rejected. One participant’s data was excluded due to excessive eye movements. The rest of the data were off-line band-pass filtered (1–30 Hz) with a finite impulse response filter. The ERPs elicited by each condition’s standard and deviant stimuli were obtained by averaging the data from each participant. The MMN for each condition was then derived by subtracting the ERP evoked by the standard stimuli from those evoked by the deviant stimuli.

Based on the grand-average waveforms obtained from the experiment and MMN time windows in previous studies [7,55,56], we chose 350–450 ms after the stimuli onset as the MMN time window for the real words’ condition and chose 200–300 ms after the stimuli onset as the MMN time window for the pseudo-words’ condition. We first took the Fz electrode as the reference point and detected the MMN peak latency in the time windows for the real and pseudo-words’ conditions. Then, based on the present experiment’s topography and MMN distributions [49,57], we selected six electrodes, F3, FC3, and C3 on the left scalp and F4, FC4, and C4 on the right scalp, and calculated the MMNs mean amplitudes with a moving time window ranging from 20 ms before the detected peak to 20 ms after that peak for each electrode.

### 2.6. Data Analysis

We conducted traditional three-way repeated-measures ANOVA and Bayes analyses via the JASP software [58] on the MMN amplitudes with lexicality (real words and pseudo-words), information (identity information, linguistic information), and hemispheres (left and right) as the within-group factors. In the Bayes analyses, we tested targeted hypotheses mentioned in the previous section (see the last paragraph in Section 1.3, The present study) using the Bayes Factor (BF), which can compute the strength of evidence for the alternative hypothesis (H1) over the null hypothesis (H0), or vice versa. Different from *p*-values (*p* > 0.05 does not provide evidence to support H0), the BFs can provide a measure of the strength of evidence for the alternative hypothesis (H1) compared with the null hypothesis (H0) [59,60,61]. Table 2 shows the classification scheme used by JASP.

### 2.7. Results

**Figure 1:**
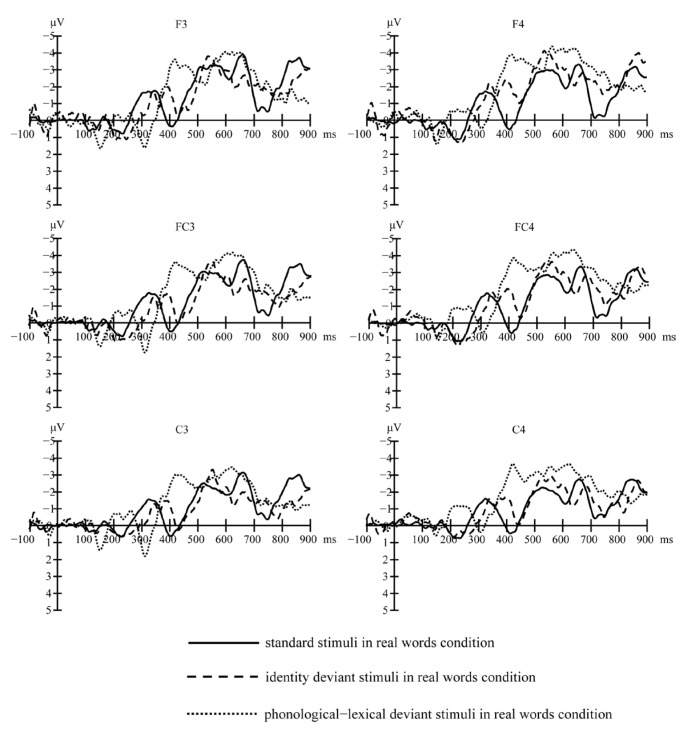
Grand-average waveforms evoked by the standard and deviant stimuli in real words’ condition at electrode locations F3, F4, FC3, FC4, C3, and C4.

**Figure 2:**
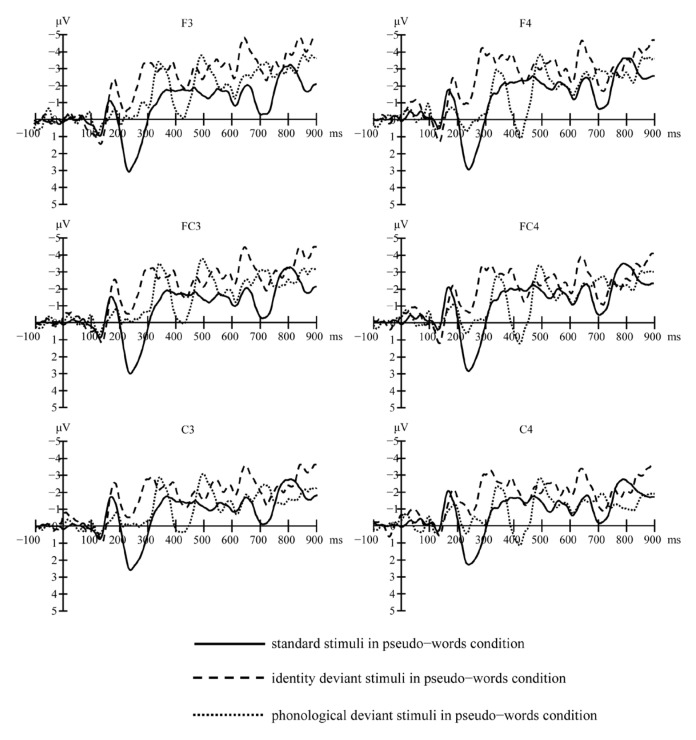
Grand-average waveforms evoked by the standard and deviant stimuli in pseudo-words’ condition at electrode locations F3, F4, FC3, FC4, C3, and C4.

**Figure 3:**
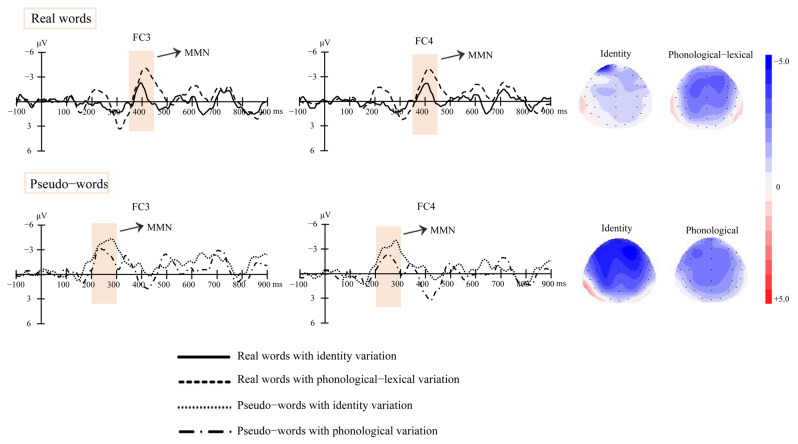
MMN waveforms evoked by the different deviant conditions at electrode locations FC3 and FC4, and the topographies of MMNs (real words: 350–450 ms; pseudo-words: 200–300 ms) in different deviant conditions.

**Figure 4:**
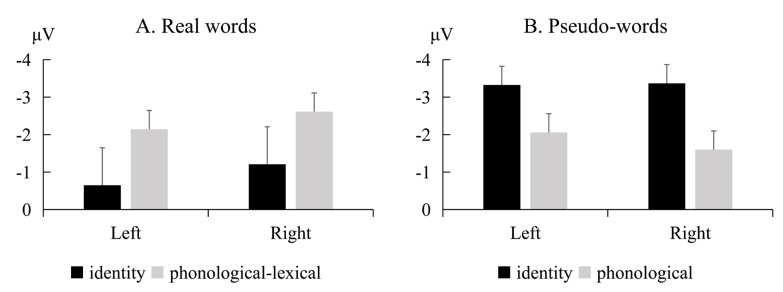
MMN amplitudes in the real words’ (**A**) and pseudo-words’ (**B**) deviant conditions. Error bars represent one standard error.

Figure 1 (real words) and Figure 2 (pseudo-words) show the grand-average waveforms evoked by the standard and deviant stimuli at the electrode locations F3, FC3, and C3 on the left scalp and F4, FC4, and C4 on the right scalp, respectively. Figure 3 shows the MMN waveforms evoked by different deviants in real words and pseudo-words on the electrodes FC3 and FC4 for examples. Figure 4 shows the MMN amplitudes in the real words’ and pseudo-words’ deviant conditions.

The ANOVA and Bayes analyses showed a significant interaction between lexicality and information (*F*(1, 22) = 8.49, *p* = 0.008, *η^2^_p_* = 0.278). The BF was 33.198, which represents very strong evidence for the alternative hypothesis. Simple effect analysis showed that for the real words’ condition, the RL’s MMN amplitude was marginally significantly larger than the RI’s MMN amplitude (*t*(22) = 2.529, *p* = 0.076, *d* = 0.556). The BF was 8.493, which can provide moderate evidence for the alternative hypothesis. Nevertheless, for the pseudo-words condition, there was no significant difference between the PI and PP’s MMN amplitudes (*t*(22) = 1.964, *p* = 0.224, *d* = 0.432). The BF was 0.757, which represents anecdotal evidence for the null hypothesis. 

The main effect of lexicality was not significant (*F*(1, 22) = 2.41, *p* = 0.135, *η^2^_p_* = 0.099). The BF was 1.048, representing anecdotal evidence for the alternative hypothesis. The main effects of information (*F*(1, 22) = 0.197, *p* = 0.662, *η^2^_p_* = 0.009, *BF* = 0.166) and hemispheres were not significant (*F*(1, 22) = 0.295, *p* = 0.593, *η^2^_p_* = 0.013, *BF* = 0.279). The BFs were between 1/10–1/3, representing moderate evidence for the null hypothesis. The interactions between lexicality and hemispheres (*F*(1, 22) = 0.671, *p* = 0.422, *η^2^_p_* = 0.03, *BF* = 0.384), and among lexicality, information, and hemispheres (*F*(1, 22) = 0.855, *p* = 0.365, *η^2^_p_* = 0.037, *BF* = 0.49), were not significant. The BFs were between 1/3–1, representing anecdotal evidence for the null hypothesis. The interactions between information and hemispheres were not significant (*F*(1, 22) = 0.469, *p* = 0.501, *η^2^_p_* = 0.021), either. The BF was 0.05, representing strong evidence for the null hypothesis.

## 3. Discussion

The present study adopted a passive oddball paradigm to examine identity and linguistic information processing at an early stage of speech perception. For neural resource involvements, we found no significant MMN amplitude differences between the identity and phonological deviants in the pseudo-words’ condition. The results suggested that the two types of information processing recruited similar neural resource involvements. However, the MMN amplitudes of phonological–lexical deviants were significantly larger than the identity deviants in the real words’ conditions. Considering the similarity between identity and phonological information, the result further indicated that lexical information processing differed from identity information processing. Listeners recruited larger neural resources in processing the lexical information than the identity information. For hemispheric lateralization patterns, we did not find MMN amplitude differences between the left and right hemispheres in the real and pseudo words’ conditions. The results indicated that the identity and linguistic information processing distributed among the whole brain and did not lateralize to a certain hemisphere. 

### 3.1. Neural Resource Involvements between the Identity and Linguistic Information Processing

The identity and phonological information vary in different acoustic features. Speakers’ voices usually differ in acoustic features like amplitude, fundamental frequency (F0), tempo, and rhythm. The phonological variations in speech, like vowels and consonants, differ in the phonetic-related acoustic features like consonantal, vocalic, diffuseness, and acuteness [12,62]. Nonetheless, the present study indicated similar neural resource involvements between the identity and phonological information processing based on the MMN amplitudes. The result suggested that, regardless of linguistic or non-linguistic, the acoustic features were processed similarly. 

The finding was consistent with the MMN findings in Tuninetti et al. (2017) and Di Dona et al. (2022) [30,31]. They found a similar MMN amplitude between identity and phonological information, indicating the similar processing between two types of information at the pre-attentive stage of speech perception. However, the two studies adopted vowels as the materials, and listeners did not have access to any information other than the identity and phonological cues that differed between each vowel. Thus, this processing similarity is reflected at the lower phoneme level. In the present study, we adopted bisyllabic words with richer phonological and lexical information and that were more aligned with natural language. The identity and phonological variations occurred at the bisyllabic word level. Our findings further indicated that the similar processing between these two types of information occurred at a word level. 

However, Di Dona et al. (2022) revealed distinct processing between the identity and phonological information at the later processing stage via LDN amplitudes [31]. LDN may reflect the auditory rule extraction processes and a transfer to long-term memory [63]. Since Di Dona et al. (2022) used rotated speech stimuli, which disrupt the natural formant structure of speech and require listeners to spend more effort to extract speech rules, they found the processing differences at the later stage [31]. In contrast, the present study adopted naturally pronounced speech stimuli that require no more effort to process, so we did not find the appearance of LDN components, which also means that processing natural speech is an easy and fast process. Another explanation for the absence of the LDN component in this study may be the materials’ differences; the late processing difference between identity and phonological information may be varied between the vowel and bisyllabic levels. Future studies could be conducted to investigate the issue.

Although Tuninetti et al. (2017) and Di Dona et al. (2022) discussed identity and phonological information processing, they did not examine the lexical information [30,31]. Knösche et al. (2002)’s early study did not differentiate the phonological from the lexical information either [32]. In the present study, we created the real vs. pseudo words’ conditions to differentiate the two types of information. The results showed that the lexical information processing recruited more neural resources than the identity information, which indicates that a higher level of speech information processing requires more neural resource involvements. 

### 3.2. Hemispheric Lateralization between the Identity vs. Linguistic Information

We also investigated the hemispheric lateralization patterns of identity and linguistic information processing. Previous views and studies indicated that identity information processing lateralized to the right hemisphere while linguistic information processing lateralized to the left hemisphere [37,39,40,41]. However, our results did not show a specific hemispheric lateralization pattern for the two types of information processing. It was inconsistent with our expected hypothesis and previous studies.

Most previous studies adopted the fMRI technique with attentive tasks, which mainly indicated attentive identity and linguistic information processing. In the present study, we adopted the passive oddball paradigm to explore the pre-attentive processing of the two types of information with no attentive bias to any information. Combining our findings with previous studies, the identity and linguistic information processing would distribute among the whole brain at an early pre-attentive stage, but lateralize to the right and left hemispheres, respectively, at a later attentive stage. Although Tuninetti et al. (2017) did not discuss the hemispheric lateralization issue explicitly, we noticed that the identity and vowel variations elicited similar MMN amplitudes between the left and right hemispheres in their study [30]. Similarly, Di Dona et al. (2022) found a topographical distribution of identity and phonological variation coherent with the MMN, being most pronounced over frontal, fronto-central, and central channels [31]. Their findings also supported the view. We considered that under the requirements of the passive oddball paradigm, listeners process the identity and linguistic information as general auditory variation at the pre-attentive stage, which performs a whole-brain processing pattern. However, when they pay more attention to the identity and linguistic information, they recruit different neural resources in the left and right hemispheres to process the information.

Nonetheless, future studies could be conducted to explore the brain network underlying identity and linguistic information processing. A recent study suggested that the brain network would be more sensitive to indicate cognitive processing than the traditional brain activation indices [64]. Detecting brain networks could be promising for explaining the different brain mechanisms between the two types of information processing. 

### 3.3. Implications for Speech Perception and Spoken Words Recognition Mechanism

The classic TRACE model mainly explained how listeners represent and process different levels of linguistic information in spoken word recognition [12]. Although it has been revised in later studies, the revised models still did not consider the identity information [7,16]. The Episodic view provided implications to take the identity information into count [22,23]. It assumed that the identity information is also represented in the mental lexicon and affects lexical access. 

Based on the TRACE model, the Episodic view, and our findings, we considered that the speakers’ identity information was also represented in the features and phonemes levels. The features level represents the acoustic features of speakers’ identities, such as amplitude, F0, and tempo. The phonemes level represents the speakers’ identities, such as the listener’s voice and his/her friends’ voices. After auditory signals are input, listeners process the different levels of identity and linguistic information according to task requirements. When attention is not required to any information in the task, the identity information processing recruits similar neural resources as the phonological information processing on the phonemes levels. However, both took fewer neural resources than the lexical information processing on the words level. Moreover, the identity, phonological, and lexical information processing did not show specific hemispheric lateralization patterns. As previous studies suggested that the identity information interacted with the linguistic information, we further hypothesized that the identity information represented on the features and phonemes levels interacted with the two levels’ traditional linguistic information. 

Moreover, our findings also have some practical implications. It can provide references for speech recognition in brain-like research and artificial intelligence. Moreover, revealing the neural mechanisms of different information processing during speech perception can also provide a theoretical basis for clinical research in patients with language disorders (e.g., phonagnosia).

In conclusion, the present study indicated that identity information processing recruited similar neural resources to phonological information processing but different neural resources from lexical information processing. The identity and linguistic information processing did not show specific hemispheric lateralization patterns at an early pre-attentive speech perception stage. The findings provide insights into speech perception and spoken word recognition mechanisms. 

## Figures and Tables

**Table 1:** The standard and deviant stimuli in each experimental condition (A and B refer to two different speakers).

Words	Information Variation	Standard Stimuli	Deviant Stimuli
Real words	Identity Phonological-lexical	A: /tong2-zhi4/(comrades) A: /tong2-zhi4/(comrades)	B: /tong2-zhi4/(comrades) A: /su4-liao4/(plastic)
Pseudo-words	Identity Phonological	A: /be2-fai4/ A: /be2-fai4/	B: /be2-fai4/ A: /fi1-tei2/

**Table 2:** A descriptive and approximate classification scheme for the interpretation of Bayes factors.

Bayes Factor	Evidence Category
>100	Extreme evidence for H1
30–100	Very strong evidence for H1
10–30	Strong evidence for H1
3–10	Moderate evidence for H1
1–3	Anecdotal evidence for H1
1	No evidence
1/3–1	Anecdotal evidence for H0
1/10–1/3	Moderate evidence for H0
1/30–1/10	Strong evidence for H0
1/100–1/30	Very strong evidence for H0
<1/100	Extreme evidence for H0

## Data Availability

The data that support the findings of this study are available on request from the corresponding authors, Yu, K. or Wang, R. The data are not publicly available due to their containing information that could compromise the privacy of the research participants.
